# SARS-CoV-2 in a tropical area of Colombia, a remarkable conversion of presymptomatic to symptomatic people impacts public health

**DOI:** 10.1186/s12879-022-07575-0

**Published:** 2022-07-26

**Authors:** Caty Martínez, Héctor Serrano-Coll, Álvaro Faccini, Verónica Contreras, Ketty Galeano, Yesica Botero, Yonairo Herrera, Alejandra Garcia, Evelin Garay, Ricardo Rivero, Héctor Contreras, Yesica López, Camilo Guzmán, Jorge Miranda, Germán Arrieta, Salim Mattar

**Affiliations:** 1 Universidad de Córdoba, Instituto de Investigaciones Biológicas del Trópico, Campus Berastegui, Córdoba, Montería, Colombia; 2 Instituto Colombiano de Medicina Tropical-Universidad CES, Medellín, Colombia

**Keywords:** Confounding factors epidemiology, Presymptomatic disease, Preventive measures, Preventive medicine and public health, Disease transmission, Infectious

## Abstract

**Background:**

The ability of SARS-CoV-2 to remain in asymptomatic individuals facilitates its dissemination and makes its control difficult. Objective. To establish a cohort of asymptomatic individuals, change to the symptomatic status, and determine the most frequent clinical manifestations.

**Methods:**

Between April 9 and August 9, 2020, molecular diagnosis of SARS-CoV-2 infection was confirmed in 154 asymptomatic people in contact with subjects diagnosed with COVID-19. Nasopharyngeal swabs were performed on these people in different hospitals in Córdoba, the Caribbean area of Colombia. The genes E, RdRp, and N were amplified with RT-qPCR. Based on the molecular results and the Cq values, the patients were subsequently followed up through telephone calls to verify their health conditions.

**Results:**

Overall, of 154 asymptomatic individuals, 103 (66.9%) remained asymptomatic, and 51 (33.1%) changed to symptomatic. The most frequent clinical manifestations in young people were anosmia and arthralgia. Adults showed cough, ageusia, and odynophagia; in the elderly were epigastralgia, dyspnea, and headache. Mortality was 8%.

**Conclusions:**

A proportion of 33% of presymptomatic individuals was found, of which four of them died. This high rate could indicate a silent transmission, contributing significantly to the epidemic associated with SARS-CoV-2.

**Supplementary Information:**

The online version contains supplementary material available at 10.1186/s12879-022-07575-0.

## Background

The SARS-CoV-2 infection has put the world’s health systems to the test to serve millions of people. In Colombia, the first case of COVID-19 was reported on March 6, 2020. In 2021, 229 million cases of SARS-CoV-2 were reported, and by that date, 1491 lineages of SARS-CoV-2 were circulating [1, 2]. The pandemic shows an epidemiological problem lies in the virus’s ability to remain in asymptomatic people. The virus’s ability increases the rate of transmissibility, preventing timely measures to contain the spread. Early identification and isolation of carriers reduce the risk of virus shedding. For this reason, it is essential to increase the screening of the asymptomatic population; unfortunately, the Colombian Ministry of Health suspended RT-qPCR tests for the detection of SARS-CoV-2 infection in this population [3].

Some reports show that about half of all SARS-CoV-2 transmissions occur before the infected individual develops symptoms [4]. However, evidence indicates that between 18% and 81% of people infected with SARS-CoV-2 never develop symptoms and, consequently, they cannot be detected by health systems. At present, the distinction between asymptomatic- presymptomatic stages can only be made mainly retrospectively [5, 6].

This work aimed to establish a cohort of asymptomatic individuals’ evolution to symptomatic ones and determine the most frequent clinical manifestations.

## Methods

Between April 9 and August 9, 2020, molecular diagnosis of SARS-CoV-2 infection was confirmed in 154 asymptomatic people in contact with subjects diagnosed with COVID-19. Oropharyngeal swabs were performed on these people in different hospitals in the department of Córdoba. The samples were submitted to the Institute for Biological Research of the Tropic of the University of Córdoba. The department of Córdoba is located in the North of Colombia, the Caribbean area, and it has 1,800,000 inhabitants. The laboratory is endorsed by the National Institute of Health (INS) to carry out the molecular tests of RT-qPCR, following the Charite of Berlin protocol, which amplifies the genes E, RdRp, and N [4]. Based on the molecular results and the Cq values, the patients were subsequently followed up through telephone calls to verify their health conditions. The follow-up of the patients was carried out monthly through a structured survey until the end of the study (Additional file 1). People were asked if they had developed symptoms related to the disease and how long they manifested them. The individuals evaluated in this study were divided into four age ranges, pediatric patients (0–17 years), young people (18–26 years), adults (27–59 years), and elderly (> 60 years, modified from World Health Organization (WHO) and American Psychological Association [7, 8]. The applied survey was subjected to a pilot test to control bias. The data supplied by the patients were verified by reviewing the epidemiological records.

The consent of the patients was obtained; they have categorized strictly anonymously. The work was endorsed by the ethics committee of the Instituto de Investigación Biológicas del Tropico (IIBT). It was carried out under international ethical standards given by the World Health Organization and the Pan American Health Organization, supported by Helsinki’s declaration, national legislation, resolution number 008430 of 1993 of the Ministry of Health of Colombia.

### Analysis of data

The data were recorded in an Excel database and analyzed using the statistical package Info-stat™ version 2018. The univariate analysis for the qualitative variables was carried out by calculating absolute and relative frequencies. The quantitative variables were calculated with measures of central tendency (median). Besides, the normality of the quantitative variables was determined by applying the Kolmogorov-Smirnov test. The bivariate analysis for the qualitative variables was performed using Pearson’s Chi-square test, and the comparison of quantitative-qualitative variables was performed using Mann Whitney U test. Besides, a principal component analysis was performed to relate the observations (age categories) and the variables (symptoms) to interpret the reciprocal relationships between observations and variables.

## Results

Overall, considering the epidemiological follow-up of 154 asymptomatic individuals, 103 (66,8%) remained asymptomatic, and 51 (33.1%) developed symptoms. The patients who remained asymptomatic were 54 women and 49 men (n = 103), and their age distribution was as follows: 16 pediatric patients (0–17 years), 20 young people (18–26 years), 49 adults (27–59 years), and 18 elderly (> 60 years).

The 51 patients who developed symptoms of the disease (presymptomatic) were 22 men (43%) and 29 women (57%). No statistically significant differences were found when comparing men and women p-value = 0.327 (Pearson’s Chi-square test). The most frequent clinical manifestations in males were dyspnea, cough, fever, and headache. The female showed fever, headache, adynamia, ageusia, odynophagia, diarrhea, and myalgia (Table 1). Regarding the clinical differences related to the sex variable, we only evidenced statistically significant differences with the presence of myalgia, which was greater among women (p-value = 0.007) (Pearson’s Chi-square test).

**Table 1:** Distribution of symptoms according to the sex

Symptoms	Male %	Female %	Total %	p-value
Headache	(10/22) 0.45	(11/29) 0.37	(21/51) 0.41	0.589
Fever	(10/22) 0.45	(10/29) 0.34	(20/51) 0.39	0.43
Anosmia	(5/22) 0.22	(8/29) 0.28	(13/51) 0.25	0.693
Cough	(7/22) 0.31	(4/29) 0.13	(11/51) 0.22	0.06
Adinamia	(3/22) 0.13	(8/29) 0.28	(11/51) 0.22	0.23
Dyspnoea	(5/22) 0.22	(5/29) 0.17	(10/51) 0.20	0.625
Diarrhea	(4/22) 0.18	(6/29) 0.21	(10/51) 0.20	0.823
Ageusia	(3/22) 0.13	(7/29) 0.24	(10/51) 0.20	0.35
Odynophagia	(3/22) 0.13	(6/29) 0.21	(9/51) 0.18	0.513
Myalgia	0	(8/29) 0.28	(8/51) 0.16	0.007*
Arthralgia	(3/22) 0.13	(5/29) 0.17	(8/51) 0.16	0.73
Pain of thorax	(1/22) 0.04	(4/29) 0.13	(5/51) 0.10	0.27
Epigastralgia	(1/22) 0.04	0	(1/51) 0.02	0.246

*Significance difference (p ≤ 0.05)

The average time of conversion from the asymptomatic to symptomatic status was between 1 and 3 days (43.1%), 4–7 days (19.6%), 8–10 days (3.9%), and between 11 and 15 days (9.9%); 23.5% did not remember the time concerning the onset of symptoms. The median Cq of asymptomatic patients was 37.35, and that of symptomatic patients was 35.24 (p-value < 0.05).

**Fig. 1:**
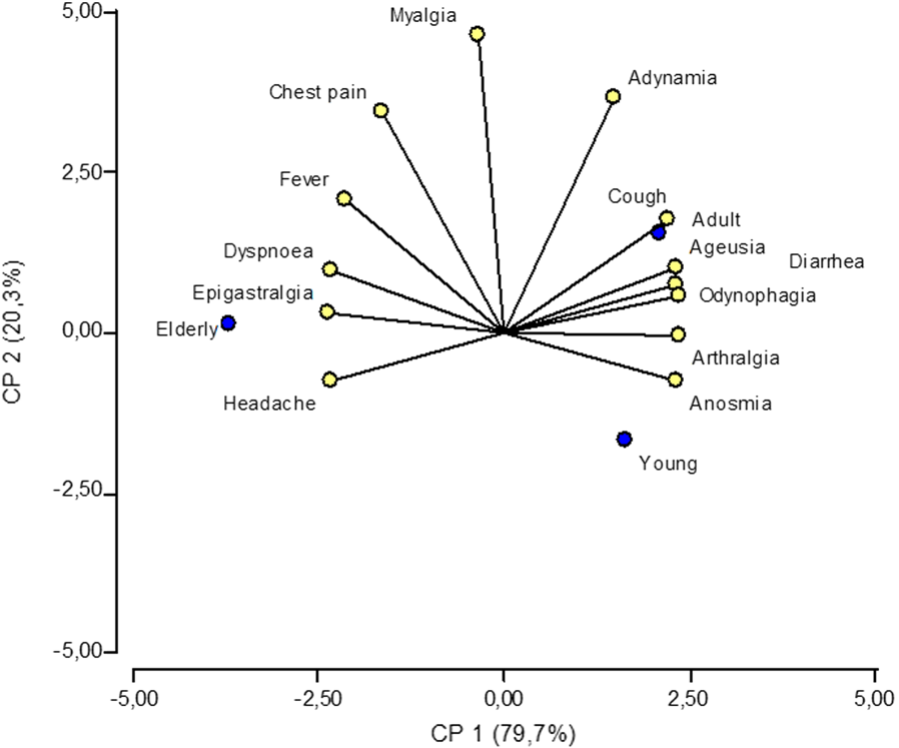
Principal component analysis of age categories and COVID-19 symptoms. The principal component, CP1, explains 79.7% of the variability of the data. The principal component, CP2, explains 20.3% of the variability of the data. CP1 and CP2 relate the main clinical manifestations for young people, adults and elderly patients

The main symptoms for all age groups were headache, fever, and anosmia (Table 2). However, we only found statistical differences in the clinical variable cough (Pearson’s chi-square test)., which was more frequent in adults (p-value = 0.041). When performing the principal component analysis for each of these groups, it was determined that in the elderly, the main manifestations were epigastric pain, dyspnea, and headache. In adults, the most common symptoms were cough and ageusia, while they were anosmia and arthralgia (Fig. 1). Additional data from the principal component analysis is available in the Additional file 2.

**Table 2:** Distribution of symptoms according to the age

Symptoms	Young^a^ %	Adult^b^ %	Elderly^c^ %	Total %	p-value
Headache	(4/10) 0.08	(10/28) 0.20	(7/13) 0.14	(21/51) 0.41	0.545
Fever	(2/10) 0.04	(10/28) 0.20	(8/13) 0.16	(20/51) 0.39	0.11
Anosmia	(3/10) 0.06	(8/28) 0.16	(2/13) 0.04	(13/51) 0.25	0.623
Cough	(2/10) 0.04	(10/28) 0.20	0	(12/51) 0.24	0.041*
Adinamia	(1/10) 0.02	(9/28) 0.18	(1/13) 0.02	(11/51) 0.22	0.127
Dyspnoea	(2/10) 0.02	(4/28) 0.08	(5/13) 0.10	(10/51) 0.20	0.134
Diarrhea	(2/10) 0.04	(6/28) 0.12	(2/13) 0.04	(10/51) 0.20	0.9
Ageusia	(2/10) 0.04	(7/28) 0.14	(1/13) 0.02	(10/51) 0.20	0.43
Odynophagia	(2/10) 0.04	(7/28) 0.14	0	(9/51) 0.18	0.145
Myalgia	(1/10) 0.02	(5/28) 0.10	(2/13) 0.04	(8/51) 0.16	0.841
Arthralgia	(2/10) 0.04	(6/28) 0.12	0	(8/51) 0.16	0.196
Pain of thorax	0	(3/28) 0.06	(2/13) 0.04	(5/51) 0.10	0.456
Epigastralgia	0	0	(1/13) 0.02	(1/51) 0.02	0.225

*Significance difference (p ≤ 0.05)

^a^Young 18–26 (included one child of 12 years old)

^b^Adult 27–59 years

^c^Elderly > 60 years

Four elderly patients who became symptomatic died, three men with comorbidities (heart disease, cancer, and kidney disease). The remaining female patient with no comorbidities also died. This last patient passed away during medical care at her home. It is important to note that deceased individuals’ Cq values ​​(median 29.93/IQR 19.6–36.2) were lower than asymptomatic and symptomatic individuals (median 37.35/IQR 35.2–38.2) and 35.24/IQR 30.6–38.9, respectively) (p-value = < 0.0001) (Mann Whitney U test).

## Discussion

In this study, it was found that 33.1% of the patients were in a presymptomatic state, and four (8%) of them died. This significant proportion could mean an eventual silent transmission, contributing to increased cases in the epidemic associated with SARS-CoV-2 [9].

Regarding the clinical manifestations related to gender, we evidence a more significant musculoskeletal commitment in the female gender in this study. This could be related to what has been published by Jeong et al. [10], who observed that musculoskeletal disorders such as myalgias are a frequent clinical manifestation in presymptomatic individuals. However, this research would infer that this clinical manifestation would have greater clinical relevance in women infected with SARS-CoV-2.

On the other hand, it is essential to mention that in developing countries such as Colombia, the diagnosis of these individuals is generally late, so they become the primary transmitters of this new virus and the perfect hosts for the generation of new variants SARS-CoV-2. On the other hand, our findings demonstrated that the most common clinical manifestations in individuals who evolved to symptoms were headache, fever, and anosmia. These findings agree with Fu et al. [11], who found that the main clinical manifestations of COVID-19 are fever, headache, cough, and dyspnea.

According to age, in the bivariate analysis, we observed that cough was a predominant clinical manifestation in presymptomatic adults, which agrees with Arons et al. [12]. They showed that cough was present in 54% of a group of presymptomatic adults. However, in the analysis of principal components, we were able to describe these presymptomatic individuals’ symptoms better, and it was observed that the most common symptoms were anosmia and arthralgia in young people. Young individuals do not seem to have significantly lower respiratory tract involvement. However, SARS-CoV-2 infection affects the upper respiratory tract, mainly causing neurological manifestations such as olfactory dysfunction, lasting up to 28 days [13]. Anosmia can be a pathognomonic clinical manifestation that could be used by healthcare personnel to differentiate SARS-CoV-2 infection from other respiratory viruses such as influenza [14]. Another study in Korea also evidenced anosmia as a frequent symptom in 3,191 young people with COVID-19 and was characteristic in mild forms of the disease [15].

The most important clinical manifestations in adults were cough, ageusia, and odynophagia, consistent with published [16]. It is striking to show that another neurological manifestation such as ageusia is one of the most frequent clinical symptoms during SARS-CoV-2 infection, which could be associated with greater involvement of the virus on the glossopharyngeal facial, and vagus nerves in this age group [17]. Therefore, ageusia could serve as a pathognomonic symptom to diagnose the adult population.

In the elderly, the clinical manifestations were epigastric pain, dyspnea, and headache. Dyspnea was in the present work a specific manifestation of older adults infected by SARS-CoV-2 [18]. Dyspnea should be considered a clinical symptom with a poor prognosis, mainly in patients with comorbidities such as hypertension, cardiovascular disease, and kidney disease [19].

Regarding the Cq values between the different clinical groups, our data are similar to those published by Quiroga et al. [20], who did not show relevant differences between the Cqs of symptomatic (37.35) and asymptomatic (35.24) individuals. In addition, it should be noted that the patients who died had a higher viral load (Cq 29.93), consistent with the work of Fajnzylber et al. [21], who showed a higher viral load induces a more significant inflammatory response and a worse prognosis. Clinical practice in those infected with SARS-CoV-2.

### Declarations

The present study has some limitations; first, we do not know how long asymptomatic individuals were infected while in contact with other individuals before diagnosis. The study also did not include data on the observed incubation period, and our data could be limited mainly to the Colombian Caribbean population, in contrast to [22]. Furthermore, the information reported here is retrospective and extrapolated mainly to the clinical behavior of SARS-CoV-2 in the year 2020.

## Conclusions

The present work demonstrates the importance of performing diagnostic tests among the contacts of individuals positive for SARS-CoV-2 since they could behave as direct disseminators of the disease. This work constitutes evidence of the evolution of asymptomatic to symptomatic patients, which contributed to monitoring signs and symptoms for cases in the Colombian Caribbean. Asymptomatic and presymptomatic subjects’ detection allowed epidemiological screening and control of the proliferation of cases. The high proportion of presymptomatic patients found in the present study is associated with significant social inequity and poor health authorities’ genomic surveillance and monitoring policy. The scarce genomic surveillance could have some epidemiological weight on the high community transmission rate of SARS-CoV-2 in the region and Colombia and the appearance of variants of interest of this new coronavirus such as Mu (B.1.621). It is essential to continue searching for asymptomatic and presymptomatic patients in Colombia. We have not yet achieved herd immunity through vaccination against SARS-CoV-2 since only 67% of the Colombian population is has completed its vaccination schedule.

Finally, it would be convenient to study the health personnel who attend COVID 19 cases despite vaccination. With the performance of tests, it would be possible to verify vaccines’ efficacy and analyze the presence and severity of symptoms.

## Supplementary Information

**Additional file 1:** Follow-up survey COVID_19 project.

**Additional file 2:** Auto vectors of the different variables associated with the principal components (PC).

## Data Availability

The datasets generated and analyzed during the current study are not publicly available but are available from the corresponding author on reasonable request.

## Abbreviations

COVID-19: Coronavirus disease 2019
SARS-CoV-2: severe acute respiratory syndrome coronavirus 2
INS: National Institute of Health
ACE2: Angiotensin-converting enzyme 2
