# Supplementary material for: SARS-CoV-2 in a tropical area of Colombia, a remarkable conversion of presymptomatic to symptomatic people impacts public health
Source: BMC Infect Dis. 2022 Jul 26;22:644. doi: 10.1186/s12879-022-07575-0 (PMC9321267; doi:10.1186/s12879-022-07575-0)
Supplement: Supplementary file 1 — Additional file 1: Follow-up survey COVID_19 project. [file 12879_2022_7575_MOESM1_ESM.pdf]

CÓDIGO IIBT:________

ENCUESTA SEGUIMIENTO PROYECTO COVID-19 2020

Buenos días, ¿hablo con [illegible] ?

Habla con *nombre del encuestador*, la Universidad de Córdoba en colaboración con el Instituto Nacional de Salud y la Secretaria de Salud Departamental estamos haciendo un estudio. ¿Nos puede regalar un par de minutos de su tiempo para responder una corta encuesta?

El día *fecha en que se tomó muestra hisopado* a usted se le tomó una muestra nasofaríngea para el diagnóstico de Covid. La Universidad de Córdoba fue la que realizó el análisis y usted salió positivo aunque no tenía síntomas.

1. Después de que le tomaron la muestra, ¿desarrolló usted algún síntoma?

-Respuesta "Si", llenar tabla **1. SÍNTOMAS.**

-Respuesta "No", pase a la pregunta **3.**

1. SINTOMAS			
1.1 DOLOR DE CABEZA		1.9 DOLOR DE GARGANTA	
1.2 CANSANCIO		1.10 DOLOR EN PECHO/TORÁCICO	
1.3 DESGANE		1.11 DIFICULTAD PARA RESPIRAR	
1.4 DOLOR MUSCULAR		1.12 DOLOR EN ARTICULACIONES	
1.5 FIEBRE		1.13 PÉRDIDA DEL GUSTO	
1.6 TOS		1.14 PÉRDIDA DEL OLFATO	
1.7 CONJUNTIVITIS		1.15 FALTA DE APETITO	
1.8 DIARREA		OTROS	

2.1 ¿A los cuántos días los comenzó a presentar?

RANGO EN DÍAS	
1-3	
4-7	
8-10	
11-15	

2.2 ¿Por cuánto tiempo?

RANGO EN DÍAS	
1-3	
4-7	
8-10	
11-15	

3. ¿Padece o padeció alguna de las siguientes condiciones? –Respuesta "Si", llenar tabla **2. ANTECEDENTES**

2. ANTECEDENTES			
2.1 PRESIÓN ALTA		2.5 OBESIDAD	
2.2 DIABETES		2.6 ENFERMEDAD DEL CORAZÓN	
2.3 ASMA		2.7 ACCIDENTE CEREBROVASCULAR	
2.4 CANCER		2.8 ENFERMEDAD RENAL	
OTROS			

4. ¿Dónde recibió los cuidados necesarios para la enfermedad? –Respuesta "Si", llenar tabla **3. DESENLACE**

3. DESENLACE			
3.1 CASA		3.4 MEJORÍA	
3.2 HOSPITALIZACIÓN		3.5 MUERTE	
3.3 UCI		OTRO	
