# Supplementary material for: SARS-CoV-2 in a tropical area of Colombia, a remarkable conversion of presymptomatic to symptomatic people impacts public health
Source: BMC Infect Dis. 2022 Jul 26;22:644. doi: 10.1186/s12879-022-07575-0 (PMC9321267; doi:10.1186/s12879-022-07575-0)
Supplement: Supplementary file 2 — Additional file 2: Auto vectors of the different variables associated with the principal components (PC). [file 12879_2022_7575_MOESM2_ESM.docx]

**Supplementary material-1.**

The principal components (PC) variances are 10.37% for PC1 and 2.63% PC2, where CP1 contributes 79.7% of the variation and CP2 20.3%. The auto vectors of the different variables associated with the components are:

| Variables | E1 | E2 |
| --- | --- | --- |
| Epigastralgia | -0,31 | 0,04 |
| Dyspnoea | -0,30 | 0,13 |
| Adynamia | 0,20 | 0,48 |
| Diarhea | 0,31 | 0,10 |
| Myalgia | -0,04 | 0,61 |
| Anosmia | 0,31 | -0,10 |
| Headache | -0,31 | -0,10 |
| Odynophagia | 0,31 | 0,07 |
| Ageusia | 0,30 | 0,13 |
| Fever | -0,28 | 0,27 |
| Cough | 0,29 | 0,23 |
| Chest pain | -0,21 | 0,45 |
| Arthralgia | -0,31 | -0,01 |
